# Fueling silence: Metabolic control of latent viral infection

**DOI:** 10.1371/journal.ppat.1014092

**Published:** 2026-04-01

**Authors:** Haixi You, Rui Guo

**Affiliations:** Department of Molecular Biology and Microbiology, Tufts University School of Medicine, Boston, Massachusetts, United States of America; University of Iowa, UNITED STATES OF AMERICA

## Altered metabolism as a foundation of viral latency

Latent viral infection is a long-term state in which the viral genome persists in host cells without production of infectious virions. In this type of infection, the virus maintains its genetic material either by integrating into the host genome or by retaining it as an extrachromosomal episome, and the viral genome is propagated through routine host DNA replication during cell division rather than through productive lytic amplification. Latent infection represents a delicate balance between genome maintenance and host defense. Many latent viruses promote cell survival, and in some cases, such as Epstein–Barr virus (EBV), this state can progress towards oncogenic transformation [1]. To sustain long-term coexistence, viruses extensively reprogram host metabolism, redirecting nutrient flux into pathways that support energy production and biosynthesis required for cell viability. Beyond fueling survival, metabolic intermediates including acetyl-CoA, S-adenosylmethionine (SAM), α-ketoglutarate, NAD, and O_2_ also act as substrates and cofactors for enzymes that write and erase epigenetic and post-translational modifications [2]. In this way, metabolic state can set the tone of latent viral chromatin and immune signaling. Importantly, stress inputs and latent viral gene products clearly contribute to latent infection as comprehensively reviewed [3–9], but here in this Pearls, we focus on how metabolic control nodes integrate those upstream cues to establish reactivation thresholds and immune evasion during latent infection. The examples discussed here are intended to illustrate emerging concepts rather than to provide a comprehensive review of latency mechanisms.

## Metabolite-mediated control of innate antiviral signaling

Pattern recognition receptors (PRRs), including RIG I-like receptors (RLRs), Toll-like receptors (TLRs), and the cytosolic DNA sensor cGAS, require adequate metabolic support for proper activation and downstream signaling, including sufficient ATP, GTP, and NAD+ [10–12]. At the same time, certain metabolites can directly modulate these pathways, either suppressing or enhancing antiviral responses [13–15]. Viral infection can actively reshape these metabolite pools and thereby influence the overall immune response. Recent studies of chronic and persistent viral infection provide direct mechanistic support for this concept. In hepatitis B virus (HBV) infection, viral induction of lactate dehydrogenase A (LDHA) increases lactate production, and the accumulated lactate binds directly to MAVS, preventing its aggregation on mitochondria and thereby inhibiting RLR-mediated interferon induction [16]. Human cytomegalovirus (HCMV) employs a distinct but conceptually related strategy. HCMV infection induces aerobic glycolysis and lactate accumulation, which in turn drives widespread lysine lactylation of host and viral proteins [17]. Tyl and colleagues found that lactyl lysine marks are enriched in intrinsically disordered regions and accumulate on key immune effectors, including the DNA sensor interferon gamma inducible protein 16 (IFI16). Specifically, K90 lactylation of IFI16 blocks recruitment of the DNA damage response kinase DNA-PK, thereby suppressing IFI16-driven viral gene repression and cytokine induction [17]. Asparagine has been recently demonstrated that directly binds TANK binding kinase 1 (TBK1) and promotes its phase separation and activation, thereby enhancing interferon responses [18]. Notably, herpes simplex virus 1 (HSV-1) counteracts this pathway by reducing expression of asparagine synthetase (ASNS), creating an asparagine-restricted state that limits TBK1 activation and helps the virus evade host innate immunity [18].

## Metabolic control of proviral silencing and reactivation in HIV

In lentiviruses such as HIV-1, latency is achieved through integrated proviral latency, where the viral genome is inserted into host chromatin and can remain transcriptionally silent in long-lived CD4^+^ T cell populations. During HIV proviral latency, cellular metabolic state is tightly coupled to the probability of proviral transcription and reactivation. The mTOR complex has been identified as a key regulator of HIV latency, where genetic or pharmacologic inhibition of mTOR signaling suppresses reversal of latency and reduces HIV transcription in multiple latency models and in cells from infected individuals [19]. Multi-omics analyses in primary CD4+ T cell infection models show that the transition to latency is accompanied by progressive downregulation of glycolysis [20]. Latently infected cells instead engage the pentose phosphate pathway. This supports NADPH production and antioxidant programs that help maintain HIV-1 latency [20]. Notably, glucose uptake can be rate-limiting for permissiveness; glucose transporter 1 (GLUT1) mediated glucose transport regulates HIV-1 infection in human CD4+ T cells and thymocytes [21]. Metabolic enzymes can also couple nutrient flux to proviral chromatin state. For example, acyl-CoA synthetase short-chain family member 2 (ACSS2) driven histone crotonylation at the HIV long terminal repeat was reported to disrupt latency and promote transcription [22].

## Methionine metabolism and methyl donor availability govern episomal chromatin states

**Fig 1:**
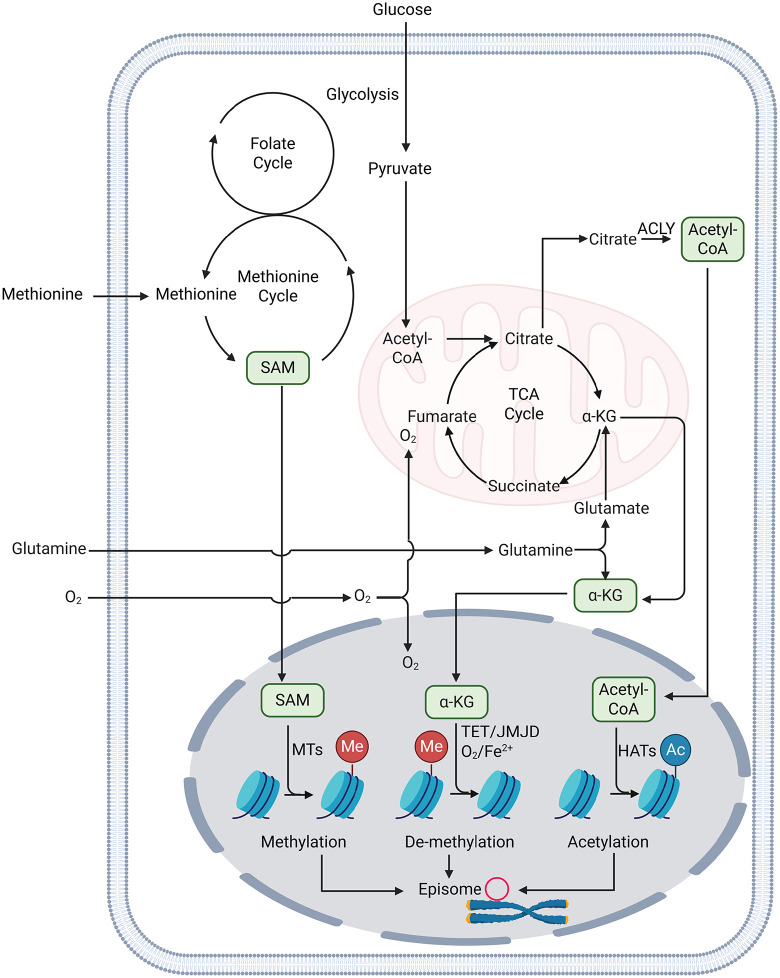
A schematic illustration depicting how metabolites influence the epigenetic regulation of latent EBV episomes. Multiple metabolic pathways exist, but only those relevant to this Pearls are shown. Abbreviations: SAM, S-adenosylmethionine; α-KG, α-ketoglutarate; TCA cycle, tricarboxylic acid cycle; MTs, methyltransferases; TET, ten-eleven translocation dioxygenases; JMJD, Jumonji C domain containing histone demethylases; HATs, histone acetyltransferases; ACLY, ATP citrate lyase; Me, methylation; Ac, acetylation. This figure was created using biorender.

Metabolism also extends its influence at the chromatin level, where it imprints durable control over viral gene expression. In EBV-infected cells, the latent episome is embedded within host chromatin and maintained through continuous input from metabolic pathways that supply cofactors for episomal chromatin modifications. Recent work has highlighted the pivotal role of the methionine cycle and its interconnected folate cycle in sustaining EBV latent genome methylation. The folate cycle supports the methionine cycle by regenerating methyl groups via 5-methyltetrahydrofolate, which donates a methyl group to homocysteine to reform methionine, ensuring a steady supply of SAM for methylation reactions [23]. Methionine is then converted by methionine adenosyltransferase into SAM, the universal methyl donor for DNA and histone methyltransferases [23]. In EBV-infected B cells, perturbation of this pathway, through methionine restriction or inhibition of folate cycle, reduces intracellular SAM, lowering methylation potential. This shift induces hypomethylation of the EBV episome, marked by loss of 5-methylcytosine (5mC) at CpG islands within latent promoters, and reactivation of lytic and latent membrane protein genes [24]. In parallel, methionine-cycle disruption also diminishes repressive histone methylation marks, notably H3K9me3 and H3K27me3, further relaxing chromatin and promoting transcriptional derepression of the viral genome [24] (Fig 1).

## Acetyl-CoA flux and histone acetylation as universal levers of herpesvirus reactivation

Histone acetylation represents a metabolically controlled axis that governs latency and reactivation across the herpesvirus family. In latently infected cell models across multiple herpesviruses, including EBV, Kaposi’s sarcoma-associated herpesvirus (KSHV), HSV-1, and HCMV, histone deacetylase inhibitors (HDACi) such as sodium butyrate and trichostatin A induce hyperacetylation of histones H3 and H4 at viral immediate-early promoters, leading to robust reactivation from latency [25–29]. This increased acetylation reflects the net activity of histone acetyltransferases (HATs) such as p300, which deposit activating acetyl marks that open chromatin and promote transcription factor access at these promoters. These findings demonstrate that the acetylation state of viral chromatin directly determines its transcriptional permissiveness (Fig 1).

In mammalian cells, the acetyl groups used for histone modification are primarily supplied by acetyl-CoA generated through ATP-citrate lyase (ACLY), which converts citrate exported from mitochondria into nucleocytosolic acetyl-CoA. Because ACLY localizes to both cytosol and nucleus, where it associates with chromatin and supports histone acetyltransferase activity, it provides the primary source of acetyl-CoA for histone acetylation under nutrient-replete conditions [30] (Fig 1). While no direct experimental evidence yet links ACLY activity to herpesviral chromatin acetylation, this pathway plausibly integrates metabolic flux with epigenetic accessibility of latent viral genomes, potentially influencing reactivation thresholds across herpesvirus infections.

## Oxygen-dependent epigenetic control of viral latency

The regulation of viral latency extends beyond intracellular metabolism. The tissue microenvironment matters. Oxygen and nutrient availability can reshape the epigenetic state of infected cells. In EBV infection, the germinal center has been proposed to play a key role in latency development [31,32]. In mouse secondary lymphoid tissues, germinal centers show low oxygen tension, with the light zone reported to be below 1% O_2_. More broadly, lymphoid tissues are typically below 3% O_2_. Limited vascularization likely contributes to this hypoxic niche [31,33]. This hypoxic condition directly impinges on oxygen-dependent chromatin enzymes, particularly the Ten-eleven translocation (TET) family of 5mC dioxygenases. Among these, TET2 requires both α-ketoglutarate, derived from the tricarboxylic acid cycle, Fe^2+^, and oxygen as essential cofactors to convert 5mC into 5-hydroxymethylcytosine (5hmC) (Fig 1). In GC B-cells, TET2 enzymatic activity is markedly reduced, leading to the accumulation of 5mC and loss of 5hmC marks across both host and viral genomes [34]. Consistent with this, Burkitt's lymphoma is thought to arise from GC B cells, and epigenetic programs established in the GC could persist after tumor cells exit that microenvironment. In EBV-positive Burkitt lymphoma, TET2 expression and activity are suppressed, and the viral episome remains densely methylated, consistent with a deeply silent latent state [35]. Recent metabolomic profiling further shows that EBV-infected B cells adapt to this GC-like environment through global metabolic remodeling, characterized by suppression of oxidative phosphorylation and fatty acid synthesis, increased reliance on glycolysis, and uptake of extracellular lipids [36]. These adaptations preserve redox homeostasis and sustain survival under low O_2_, but they also reduce NAD⁺ and acetyl-CoA availability, thereby limiting the activity of histone acetyltransferases [2,36].

Histone methylation provides a second O_2_-sensitive epigenetic axis that can reinforce latency in parallel with DNA methylation changes. Work in HSV-1 latency established a foundational model in which lytic gene promoters on latent viral genomes are enriched for heterochromatin marks including H3K27me3 and H3K9me3, restricting immediate early gene expression during latency [37,38]. Reactivation requires removal of these repressive histone methylation marks by histone demethylases, and inhibition of these enzymes blocks α-herpesvirus lytic replication and reactivation [38–40]. Interestingly, many of the relevant demethylases belong to the Jumonji C domain containing family, which are Fe^2+^ and α-ketoglutarate-dependent dioxygenases that also require O_2_ for catalysis. As O_2_ levels drop, demethylase activity decline, shifting chromatin toward higher methylation states even without changes in transcription factor inputs [41]. In EBV latency, H3K27me3 is likewise a core repressive mark at the immediate early lytic promoters, specifically the *BZLF1* (*Zp*) and *BRLF1* (*Rp*) promoters that encode the Zta and Rta transcription factors that initiate lytic cycle entry [42]. This repression is potentially reinforced in hypoxic microenvironments like GC. The major H3K27me3 demethylases KDM6A and KDM6B are JMJDs, and KDM6A has been shown to directly sense O_2_, with hypoxia reducing its H3K27 demethylation capacity and increasing H3K27 methylation independently of HIF signaling [43]. This could potentially favor maintenance of a repressive H3K27 methylation state on the herpeviral episomes.

Interestingly, GC-like hypoxia also stabilizes HIF-1α, a transcription factor capable of activating the immediate-early promoters of EBV and KSHV to initiate the lytic cycle in cell culture models [44,45]. At first glance, this seems paradoxical, because the GC microenvironment is otherwise strongly repressive to viral gene expression. This contradiction can be resolved by a model in which HIF-1α-driven lytic activation is counterbalanced, and ultimately overridden, by epigenetic and metabolic constraints imposed by the GC niche *in vivo*. Within the hypoxic and nutrient-limited GCs, viral promoters acquire DNA methylation and are packaged into compact chromatin. Simultaneously, limited respiration and TCA cycle reactions restrict the energetic and biosynthetic capacity required to support full viral lytic replication. As a result, even if HIF-1α transiently engages and activates lytic promoters, its transactivation potential can be curtailed by the combined effects of metabolic insufficiency and repressive chromatin architecture. When infected B cells differentiate into memory B cells and exit the GC into normoxic circulation, HIF-1α is destabilized, removing this acute pro-lytic stimulus. However, the heavily methylated, transcriptionally silent viral episome established during GC residency persists. Maintenance factors such as UHRF1, which recruits DNMT1 to hemi-methylated CpG sites during DNA replication, ensure that these repressive methylation patterns on the latent episome are faithfully propagated through subsequent cell divisions [46].

## Metabolic control of adaptive immunity in latent viral persistence

**Fig 2:**
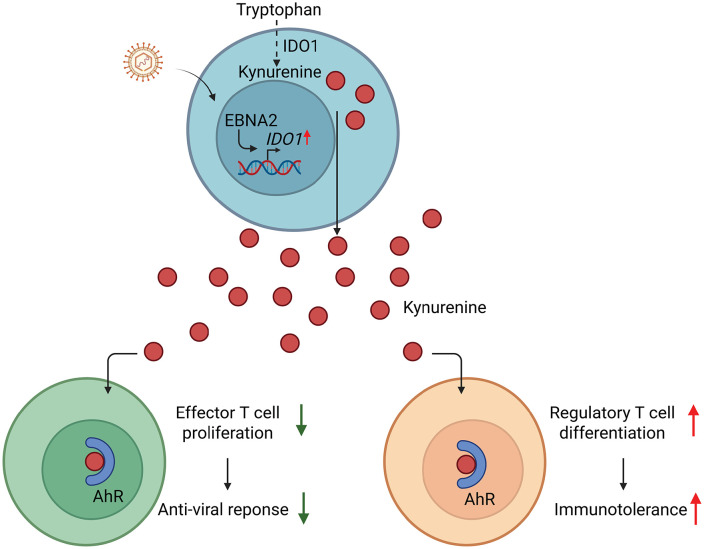
A schematic illustration showing how virus-induced tryptophan catabolism elevates kynurenine levels, leading to suppression of effector T cells and activation of regulatory T cells (Tregs), thereby promoting immunotolerance during long-term infection in vivo. Abbreviations: IDO1, indoleamine 2,3 dioxygenase 1; EBNA2, Epstein–Barr virus nuclear antigen 2; AhR, aryl hydrocarbon receptor. This figure was created using biorender.

Latent viruses also reshape the adaptive immune system by exploiting metabolic checkpoints that govern T-cell function. T lymphocytes rely on distinct metabolic programs to support their effector versus regulatory states: activated effector T cells depend on aerobic glycolysis to sustain proliferation and cytokine production, whereas regulatory T cells (Tregs) and long-lived memory T cells rely predominantly on fatty acid oxidation and oxidative phosphorylation [47]. Viral manipulation of host metabolism has been shown to skew the balance of T-cell subsets and blunt antiviral immunity. For example, EBV nuclear antigen 2 (EBNA2) induces the expression of indoleamine 2,3-dioxygenase 1 (IDO1), a rate-limiting enzyme driving tryptophan catabolism toward kynurenine production [48,49]. Kynurenine is an established ligand for the aryl hydrocarbon receptor (AhR), a transcription factor that promotes Treg differentiation and suppresses effector T-cell proliferation [50,51]. Of interest, conditioned medium from EBV-infected cells restricted T cell expansion through IDO1-mediated tryptophan depletion, while kynurenine accumulation dampened antiviral T cell activity through AhR engagement, thereby promoting an immunosuppressive niche favorable for viral persistence [48] (Fig 2). However, additional in vivo models will be needed to define how broadly this mechanism operates during latent infection.

## Concluding remarks

Latent viruses exploit host metabolism not simply to support their own replication, but to hard-wire immune evasion into the cell’s biochemical circuitry. Across latent viruses, three principles consistently emerge: (1) metabolic state dictates the strength of innate signaling through direct modification of sensors and adaptors; (2) metabolite availability determines the epigenetic accessibility of latent viral genomes; and (3) metabolic checkpoints shape the differentiation and functional capacity of antiviral T cells. These mechanisms converge on a shared strategy in which viruses avoid detection not by remaining inert, but by actively steering metabolic fluxes into pathways that suppress sensing, restrict chromatin accessibility, and bias adaptive immunity toward tolerance. Key open questions include (1) how latent viral factors remodel nutrient landscapes, and whether this remodeling feedback to fortify latency, (2) whether viral episomes retain metabolite dependent epigenetic memory of past stress that biases future reactivation, (3) which host metabolite transporters act as dominant gatekeepers of latency, and (4) whether infected cells generate local immunosuppressive metabolite gradients at the neighborhood. Answering these questions may reveal leverage points where targeting host metabolic dependencies, rather than viral proteins alone, destabilizes latency or restores antiviral immunity.
